# What is your diagnosis?

**DOI:** 10.4274/jtgga.2018.0132

**Published:** 2018-11-15

**Authors:** Banu Seven Yüksel, Nafiye Yılmaz, Evin Nil Uğurlu, Cavidan Gülerman, Yaprak Engin-Üstün

**Affiliations:** 1 Department of Obstetrics and Gynecology, University of Health Sciences, Ankara Dr. Zekai Tahir Burak Women Disease Training and Research Hospital, Ankara, Turkey

A 25-year-old woman with unexplained infertility was admitted to our assisted reproductive technology clinic. Her fertility assessment was found to be normal. On her third menstrual day, the total antral follicle count was 15, anti-mullerian hormone level was 4.8 ng/mL, follicle-stimulating hormone level was 9 mIU/mL, and the estradiol level was 16 pg/mL. She had 4 previous in vitro fertilization attempts at various centers. In her first two cycles, no mature oocytes (M2) were revealed. Although 9 oocytes were collected in her second cycle, none was an M2 oocyte. In her third cycle, 13 oocytes were collected and 6 were M2. Only 1 was fertilized and embryo transfer was performed on the third day. In her fourth cycle, a dual trigger, consisting of a gonadotrophin-releasing hormone analogue and human chorionic gonadotrophin, was applied for the final oocyte maturation and ovulation trigger. A total of 19 oocytes were collected and none was M2; however, fertilization did not occur.

In her fifth cycle at our clinic, we initially checked all her previous fertility assessments, ovulation induction protocols, triggers administered, and reproductive outcomes. In light of her previous reports, we chose an antagonist protocol with 300 IU human menopausal gonadotrophin/day and applied a ‘dual trigger’. The peak estradiol level on the oocyte trigger day was 1696 pg/mL and the total gonadotrophin dosage was 2400 IU. Eleven oocytes were collected, 1was M2, but fertilization did not ensue.

## Answer

Successful fertilization and embryonic development in humans needs union of sperm and a mature oocyte. For maturation, the oocyte has to undergo some changes including growth, and mRNA and proteins accumulation (1). During embryonic development, oocytes first undergo meiotic progression and become arrested in the diplotene stage of prophase I at the time of birth. Upon a surge in luteinizing hormone (LH) just before ovulation, oocytes resume meiosis and progress through the second meiotic cycle and arrest at metaphase II until fertilization, this is called oocyte maturation. Attaining this molecular competence requires multiple factors regulated by different signaling pathways.

Oocyte maturation arrest (OMA) is presumed to be due to inadequate LH activity, the defect in signaling mechanism surrounding cumulus cells or intrinsic oocyte factors (2). Although the exact mechanisms and causes of this disorder are not known, a genetic deficiency of regulatory proteins or genetic alterations of genes or protein expressions of regulatory proteins and enzymes, or oocyte-specific alterations of transcription factors may contribute (1,2). There are some treatment strategies as dual-trigger and double-trigger applications, but a definitive treatment modality is lacking; however, investigating the genetic basis of OMA will provide great insight into understanding the mechanisms of this disorder, as well as improving treatment strategies.

Although we reviewed all previous cycle reports and changed the ovulation induction protocol to an antagonist protocol co-treated with a dual trigger, we obtained only 1 mature oocyte out of 11.

We performed genetic testing in view of its possible genetic association. Thirty-six genes related with reproductive functions were analyzed and heterozygote mutations in PROKR2 p. T273M, FSH p. Ala307Thr and p. Ser680Asp genes were identified. Mutations in FSH p. Ala307Thr and p. Ser680Asp genes have been reported to be associated with poor ovarian reserve, and mutations in PROK2 genes were linked to hypogonadotropic hypogonadism (3), but the exact pathogenic mechanisms of these mutated genes and any association with OMA have not been elucidated thus far. In the literature, data regarding that subject is scarce and subject to investigation.

These findings may suggest some roles for these mutations in oocyte maturation arrest and expand our knowledge in terms of the genetic basis of female infertility. Unraveling molecular and genetic basis of OMA will help patients by improving diagnosis and our understanding of the disease. This will guide us in counseling patients about treatment outcomes, develop strategies to overcome this disorder, and allow for better informed decisions regarding treatment options and prevent unnecessary interventions.
